# Managing Obesity and Related Comorbidities: A Potential Pharmacological Target in the Adenosine System?

**DOI:** 10.3389/fphar.2020.621955

**Published:** 2021-01-18

**Authors:** Vanessa D’Antongiovanni, Matteo Fornai, Carolina Pellegrini, Corrado Blandizzi, Luca Antonioli

**Affiliations:** ^1^ Department of Clinical and Experimental Medicine, University of Pisa, Pisa, Italy; ^2^ Department of Pharmacy, University of Pisa, Pisa, Italy

**Keywords:** adenosine, adenosine receptors, obesity, adipogenesis and lipogenesis, diabete mellitus, cardiovascular diseases, gastrointestinal dysfunction, cognitive impairment

## Introduction

Obesity is a complex and multifactorial disease characterized by abnormal fat accumulation resulting from a disequilibrium between energy intake and its consumption (Bluher, 2019). At present, more than one-third of the world population is classified as obese or overweight, and according to a recent estimation it is expected that by 2030 this value will surpass 50% (Bluher, 2019).

Obesity is associated with a condition of low-grade systemic inflammation that seems to be a common root to the onset and progression of several comorbidities, such as type-2 diabetes mellitus (T2DM), cardiovascular diseases (CVDs) and cognitive impairment (Jarolimova et al., 2013). In parallel, there is evidence that obese patients often complain of chronic gastrointestinal (GI) disturbances, including gastroesophageal reflux, diarrhea and constipation, undermining their quality of life (Camilleri et al., 2017; D'Antongiovanni et al., 2020a).

Preclinical studies on mice fed with high-fat diet (HFD) have shown that the adipocytes and adipose tissue-associated macrophages release a plethora of inflammatory mediators, including interleukin (IL)-1, IL-6, tumor necrosis factor (TNF) and monocyte chemoattractant protein-1, leading to a condition of systemic inflammation and oxidative stress (Ellulu et al., 2017). The chronicization of this inflammatory condition then affects the homeostatic mechanisms, and thereby the physiological functions, of several organs, leading to the development of different obesity-associated comorbidities (Ellulu et al., 2017). At present, the available pharmacological tools to manage obesity are unsatisfactory in terms of efficacy, safety and long-term maintenance of weight loss. Therefore, the identification of novel molecular targets, aimed at developing innovative anti-obesity treatments, represents a challenging and exciting field of high scientific interest.

Over the years, several lines of evidence have outlined a critical role of the adenosine system in glucose homeostasis, inflammation, adipogenesis, insulin resistance and thermogenesis, thus suggesting an involvement of adenosine in the onset and progression of obesity (Pardo et al., 2017; D’Antongiovanni et al., 2020b). Indeed, the wide distribution of adenosine receptors (ARs) in tissues tightly involved in metabolism regulation leads to hypothesize the pharmacological modulation of adenosine pathways as a viable way to counteract obesity and related comorbidities (Hasko et al., 2008; Antonioli et al., 2011; Kotanska et al., 2020). In line with this view, encouraging preclinical data have reported beneficial effects of treatments with specific AR ligands in stemming adipose tissue inflammation and insulin resistance (Figler et al., 2011; Csoka et al., 2014).

Based on the above background, the present opinion paper has been conceived to provide a critical appraisal of the available knowledge about the involvement of adenosine system in the pathophysiological mechanisms underlying obesity, pointing out its potential as therapeutic target to develop innovative therapeutic strategies aimed at counteracting the obesity-associated diseases.

## adenosine system: enzymes, transporters, receptors and physiological functions

**FIGURE 1:**
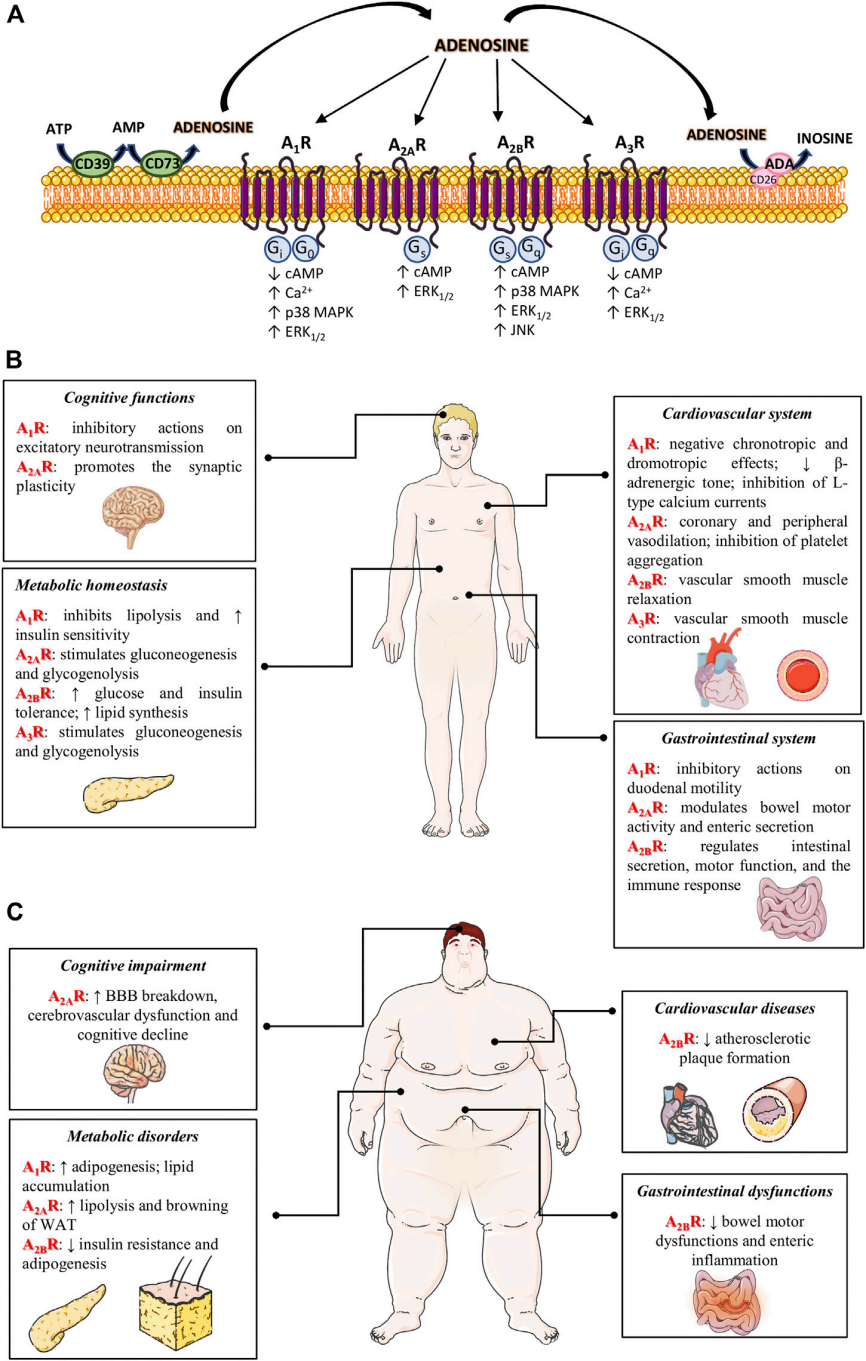
**(A)** Adenosine receptor signaling. The extracellular adenosine is mainly produced by the ectonucleotidases, CD39 and CD73. Excess adenosine is irreversibly deaminated to inosine by ADA. Adenosine can bind to four different G protein-coupled adenosine receptors, that either inhibit (A_1_R and A_3_R) or stimulate (A_2A_R and A_2B_R) adenylyl cyclase activity and cAMP production in the cell. All ARs are coupled also to MAPK pathways, including ERK_1/2_ and p38 MAPK. **(B)** Actions of adenosine receptors on various organ under physiological conditions. In CNS, A_1_Rs exert inhibitory actions on excitatory neurotransmission, while A_2A_Rs promote excitatory actions. In cardiovascular system, the engagement of A_1_Rs and A_3_Rs triggers the vascular smooth muscle contraction, whereas the activation of A_2A_Rs and A_2B_Rs mediates the vascular smooth muscle relaxation. In addition, A_1_R activation triggers negative chronotropic and dromotropic effects and A_2A_R engagement inhibits platelet aggregation. In gastrointestinal system, the activation of A_1_Rs, A_2A_Rs and A_2B_Rs regulates intestinal secretion and motor function. Furthermore, the activation of A_2A_Rs and A_3_Rs stimulates gluconeogenesis and glycogenolysis; while, A_1_Rs and A_2B_Rs inhibit lipolysis and increase the lipid synthesis. **(C)** Actions of adenosine receptors on obesity and related comorbidities. The activation of A_1_Rs stimulates adipogenesis and lipid accumulation, thus promoting weight gain. By contrast, the activation of A_2B_Rs reduces adipogenesis, insulin resistance and atherosclerotic plaque formation; it counteracts also enteric inflammation and gastrointestinal dysfunction. The engagement of A_2A_Rs triggers browning process and lipolysis, and mediates obesity-induced BBB breakdown and cognitive dysfunctions. ↑, increase; ↓, decrease. Abbreviations: *A*_*1*_ *R*, adenosine 1 receptor; *A*_*2A*_ *R*, adenosine 2A receptor; *A*_*2B*_ *R*, adenosine 2B receptor; *A*_*3*_ *R*, adenosine 3 receptor; *ADA*, adenosine deaminase; *ATP*, adenosine triphosphate; *BBB*, blood-brain barrier; *Ca* ^*2+*^, calcium ions; *cAMP*, cyclic AMP; *CNS*, central nervous system; *ERK*_*1/2*_, extracellular signal-regulated kinase 1/2; *JNK*, JUN N-terminal kinase; *MAPK*, mitogen-activated protein kinase; *WAT*, white adipose tissue.

Adenosine is an endogenous nucleoside derived from the metabolism of adenosine triphosphate (ATP) (Sheth et al., 2014). Physiologically, adenosine is present at low levels in the interstitial fluids of unstressed tissues, with its concentration increasing quickly in the presence of metabolically stressful conditions (D’Antongiovanni et al., 2020b). Intracellular adenosine is produced from S-adenosylhomocysteine via S-adenosylhomocysteine hydrolase (Sheth et al., 2014). In the extracellular space, adenosine results mainly by the dephosphorylation of ATP, mediated in a sequential manner by ecto-nucleotide triphosphate diphosphohydrolase-1 (also named CD39) and by ecto-5′-nucleotidase (also named CD73) (Eltzschig, 2013; Antonioli et al., 2013) (Figure 1A). The extracellular and intracellular adenosine levels are finely tuned by the activity of the nucleoside transporters classified into: equilibrative nucleoside transporters (ENTs), which transport nucleosides across cell membranes in either directions, and concentrative nucleoside transporters (CNTs), which shunt extracellular adenosine into the intracellular space against their concentration gradient (Pastor-Anglada et al., 2018). Another critical checkpoint in the regulation of adenosine levels is represented by the adenosine deaminase (ADA), a key enzyme involved in degradation of adenosine into inosine (Antonioli et al., 2012) (Figure 1A).

Most of the biological actions of adenosine are mediated by four different subtypes of G-protein coupled receptors, that either inhibit (A_1_R and A_3_R) or stimulate (A_2A_R and A_2B_R) adenylyl cyclase activity and cAMP production in the cell. All ARs are coupled also to MAPK pathways, including ERK_1/2_ and p38 MAPK (Sheth et al., 2014) (Figure 1A). In addition, adenosine can also exerts receptor-independent effects, via less defined intracellular mechanisms, including the S-adenosylhomocysteine hydrolase systems, adenosine kinase and AMP-activated protein kinase (AMPK) (Da Silva et al., 2006; Boison, 2013). Once released into the extracellular space, adenosine takes a pivotal part in modulating a wide variety of physiological processes, including gut motility, blood flow, lipolysis and gluconeogenesis (Figure 1B).

## ROLE OF ADENOSINE IN ADIPOGENESIS AND LIPOLYSIS

Adipogenesis is a physiological process of cell differentiation by which preadipocytes differentiate into adipocytes, with a consequent increase in the formation of white adipose tissue (WAT) (Ouchi et al., 2011). An abnormal increment of WAT results in an increased release of pro-inflammatory cytokines along with altered adipokine secretion (Ouchi et al., 2011). In this context, adenosine, released continuously from adipocytes, is able to promote adipogenesis and lipid accumulation via A_1_R (Gharibi et al., 2012; Burnstock and Gentile, 2018). By contrast, the engagement of A_2B_Rs is followed by the inhibition of preadipocyte differentiation, indicating a differential role of adenosine on adipogenesis, based on the receptor subtype engaged (Eisenstein et al., 2014).

It is worth to note that ARs play also important roles in controlling the functions of brown adipocytes. The brown adipose tissue (BAT), critically involved in thermogenesis and lipolysis, is emerging as a potential target for anti-obesity therapies (Cully, 2014). It is well acknowledged that A_1_R activation can blunt the lipolysis of brown adipocytes in rodents (Mohell, 1984). However, recent studies allowed to observe that A_2A_Rs, expressed mainly on brown adipocytes, are involved markedly in the process of thermogenesis (Cully, 2014; Gnad et al., 2014). In this regard, Gnad et al. observed that the pharmacological inhibition of A_2A_Rs, by its antagonist MSX-3, as well as the genetic deletion of these receptors in mice induced a decrease in BAT-dependent thermogenesis (Gnad et al., 2014). By contrast, treatment with A_2A_R agonist, CGS21680, significantly increased energy expenditure, demonstrating the relevant contribution of A_2A_R in mediating the thermogenic response (Gnad et al., 2014). In the same study, the authors also showed that the systemic administration of CGS21680, beyond to reduce the body weight gain, induced the browning of WAT and improved glucose tolerance in HFD mice (Gnad et al., 2014). Taken together, these results demonstrated that A_2A_R activation triggers lipolysis, energy expenditure and browning of WAT, indicating a beneficial effect in counteracting the HFD-induced obesity in mice.

## Obesity-Related Comorbidities: Role of Adenosine

### Type-2 Diabetes Mellitus

T2DM is a pathological condition, often associated with obesity, characterized by impairment of insulin secretion and/or function (Hardy et al., 2012). In obese patients, the underlying chronic low-grade inflammatory condition represents a pivotal factor in the onset of insulin resistance, a prodromal step leading to the development of T2DM (Nowotny et al., 2015). As a consequence of insulin resistance, a compensatory hyperinsulinemia occurs, with an over-stimulation of pancreatic β-cell function, followed by their exhaustion (Nowotny et al., 2015).

Several studies have reported a relationship between the adenosine system and obesity-induced insulin resistance and T2DM. Some authors observed that the over-expression of A_1_Rs in the adipose tissue of knock-in mice protected from HFD-induced insulin resistance, indicating this receptor subtype as a potential therapeutic target for the management of obesity-related insulin resistance and T2DM (Dong et al., 2001). However, it is noteworthy the A_2B_R subtype seems to play also pivotal roles in modulating glucose homeostasis and insulin resistance, thus emerging as a promising target for drug development. Indeed, the systemic administration of the selective A_2B_R agonist, BAY60-6583, reduced significantly the plasma glucose, insulin and IL-6 levels, and ameliorated T2DM in HFD mice (Johnston-Cox et al., 2014).

Overall, current data provide evidence that targeting A_1_R or A_2B_R activation could represent a useful therapeutic strategy for preventing and/or treating obesity-related metabolic disorders.

### Cardiovascular Diseases

Obesity is associated with an increased risk of developing CVDs, including hypertension, atherosclerosis and myocardial infarction (Carbone et al., 2019). Increasing evidence suggests that the excess of adipose tissue favors the secretion of pro-inflammatory cytokines and an overproduction of oxidant molecular species that affect the cardiovascular system directly or indirectly (Carbone et al., 2019).

Over the last years, the pharmacological modulation of ARs has emerged as a promising therapeutic approach also for the management of CVDs (Jacobson et al., 2019). In this regard, it has been reported that the activation of myocardial A_1_Rs by the partial agonist capadenoson protects from ischemia-reperfusion injury in a rat model of acute myocardial infarction, thus corroborating the evidence about a cardioprotective role of this receptor subtype (Albrecht-Kupper et al., 2012). Other authors observed an anti-hypertensive effect after treatment with the A_2A_R agonist CGS21680 in a mouse model of hypertension induced by partial constriction of the renal artery, highlighting a modulatory role of A_2A_Rs in regulating vascular smooth muscle relaxation/contraction (Schindler et al., 2005). Despite these interesting findings, currently only one preclinical study has investigated the effects of AR ligands on CVDs associated with obesity. In this setting, Koupenova et al. reported a beneficial effect of the A_2B_R agonist BAY60-6583 on atherosclerotic plaque formation in HFD mice as a consequence of a decrease in cholesterol and triglyceride plasma levels (Koupenova et al., 2012). Despite the need for additional studies, this preliminary evidence suggests the pharmacological modulation of A_2B_R as an intriguing strategy for the therapeutic management of CVDs associated with obesity.

### Gastrointestinal Dysfunctions

Obesity is characterized by GI disturbances, including gastroesophageal reflux, irritable bowel syndrome, diarrhea and constipation (Camilleri et al., 2017; D'Antongiovanni et al., 2020a). Preclinical studies have shown that HFD animals, beyond showing alterations of gut microbiota and intestinal epithelial barrier, present a low-grade enteric inflammation, which contributes to the onset of intestinal dysfunctions associated with obesity (Bhattarai et al., 2016; Antonioli et al., 2017; Antonioli et al., 2020).

Increasing evidence highlights a critical role for the adenosine system in the pathophysiology of bowel dysfunctions associated with obesity. In particular, Antonioli et al. observed that adenosine, via A_2B_Rs, participates to obesity-related enteric dysmotility, modulating the activity of excitatory tachykininergic nerves in HFD mice (Antonioli et al., 2017). In support of this finding, a recent study confirmed the contribution of A_2B_Rs, expressed on enteric glial cells, in the modulation of tachykininergic responses and enteric inflammation associated with obesity, thus corroborating the relevant contribution of A_2B_Rs to the pathophysiology of bowel motor dysfunctions and inflammation associated with obesity (D’Antongiovanni et al., 2020c). Based on these data, the pharmacological modulation of A_2B_R represents a viable way to design novel tools to manage both bowel motor dysfunctions and enteric inflammation associated with obesity.

### Cognitive Impairment

Increasing evidence support the concept that obesity can lead to neuroinflammation, and neurodegenerative diseases (i.e., Alzheimer’s disease), and can affect negatively cognition, including attention and decision making (Leigh and Morris, 2020). In line with this view, obese mice display brain dysfunctions and learning impairment (Leigh and Morris, 2020).

Adenosine has been shown to play a critical role in the control of the cognitive functions (Choudhury et al., 2019). In particular, adenosine was found to exert both inhibitory actions on excitatory neurotransmission, via A_1_R activation, and excitatory actions driven by engagement of A_2A_Rs (Gomes et al., 2011). In the context of obesity, a limited number of experimental studies have been focused on the possible involvement of ARs in the pathophysiology of cognitive impairment. For instance, Yamamoto et al. investigated the role of A_2A_Rs in obesity-induced cognitive dysfunctions. They described an increase in blood-brain barrier (BBB) breakdown along with cognitive impairment in HFD mice, and observed that the genetic ablation of A_2A_Rs in endothelial cells protected HFD mice against the BBB impairment and cognitive dysfunction (Yamamoto et al., 2019). This study provides the first evidence that changes in cerebrovascular permeability initiate the cycle of obesity-induced neuroinflammation and cognitive impairment. In this context, the pharmacological modulation of A_2A_Rs could be considered as an interesting molecular target to design novel therapeutic strategies aimed at managing the cognitive impairment associated with obesity.

## Conclusion

Nowadays, the therapeutic management of obese patients remains unsatisfactory. A body of preclinical knowledge indicates that the pharmacological modulation of adenosine system has a promising potential for treating obesity and related comorbidities. In this regard, the development of experimental models of obesity, including diet-induced models as well as the genetic models (i.e. leptin-deficient model) allowed to better understand the role of adenosine system in the pathophysiological mechanisms underlying obesity. Indeed, adenosine modulates actively glucose homeostasis, inflammation, adipogenesis, insulin resistance and thermogenesis, depending on the engagement of receptor subtypes in different tissues (Fredholm, 2014). For instance, A_1_R activation stimulates adipogenesis and lipid accumulation, thus promoting weight gain. Accordingly, future efforts should be focused on investigating better the effects of A_1_R ligands on lipogenic and thermogenic processes in the context of obesity. The activation of A_2A_Rs can trigger the browning process of WAT, support the lipolytic process, and counteract significantly the obesity-induced BBB alterations and cognitive dysfunctions. Along the same line, the pharmacological stimulation of A_2B_Rs can exert beneficial effects on obesity, since these receptors participate actively in shaping adipogenesis, insulin resistance, inflammation, atherosclerotic plaque formation and GI dysfunctions associated with obesity (Figure 1C). However, the paucity of selective A_2B_R ligands limits greatly the exploration of the therapeutic implications of these receptor subtypes. Therefore, the synthesis of highly selective compounds is needed, particularly to curb the occurrence of adverse effects. At present, no data are available about the involvement of A_3_Rs in the onset and development of obesity-associated comorbidities.

Despite current evidences arising from the animal models are encouraging, the translation of preclinical data into clinical practice will require a more thorough understanding of the tissue-specific effects of adenosine. At present, the main point of weakness concerns the limited methods to quantify the adenosine and ADA activity *in vivo*.

Over the years, a growing body of preclinical evidence has highlighted the possibility of beneficial effects resulting from the pharmacological modulation of adenosine pathways in the context of obesity. However, a number of issues regarding the regulatory role of digestive functions by the adenosine system are still pending and deserve further investigations. Of note, the available clinical data regarding the involvement of the adenosine system in obesity are scanty, and it is not possible to have consolidated evidence or draw substantial conclusions.

Taken together, the pharmacological modulation of the adenosine system represents an attractive strategy for the scientific community, encouraging the development of novel AR ligands useful to manage and counteract obesity and its related comorbidities.

## Author Contributions

VD and LA literature search, writing original draft preparation; CP helped in the preparation of the figures, and reviewed the draft; MF and CB reviewed the draft. All authors have read and agreed to the published version of the manuscript.

## Conflict of Interest

The authors declare that the research was conducted in the absence of any commercial or financial relationships that could be construed as a potential conflict of interest.
